# Why Is Chromic Acid Such a Valuable Caustic?

**Published:** 1884-05

**Authors:** 


WHY IS CHROMIC ACID SUCH A VALUABLE CAUSTIC?—Dr. Squibb answers: "Because it is self-limiting in its action in a degree that no other destructive caustic is. It is an active oxidizing agent, and destroys the tissues to which it is applied by oxidation. In this respect it is like other caustics, as nitric acid. But every molecule of chromic acid which destroys a molecule of organic tissue is itself destroyed and rendered inert by being reduced to an insoluble oxide of chromium; and this principle and degree of self-limitation is not obtained from any other caustic.—*Detroit Lancet.*

